# Association between uncertainty stress and self-rated health among Chinese healthcare professionals: a nationwide cross-sectional survey

**DOI:** 10.3389/fpubh.2026.1811271

**Published:** 2026-06-04

**Authors:** Lihong Wu, Jianhua Chen, Jianjiang Pan, Jingjing Xia, Liying Chen

**Affiliations:** Department of General Practice, Sir Run Run Shaw Hospital, School of Medicine, Zhejiang University, Hangzhou, Zhejiang, China

**Keywords:** cross-sectional study, healthcare professionals, occupational stress, self-rated health, uncertainty stress

## Abstract

**Background:**

Research on the association between uncertainty stress (US) and overall health status among healthcare professionals (HCPs) remains limited. Thus, this national cross-sectional study aimed to examine the relationship between US and self-rated health (SRH) in this population.

**Methods:**

A cross-sectional online survey of 2,976 HCPs was conducted from September 2022 to January 2023 across three Chinese provinces (eastern, central, western). A two-stage sampling approach was used: purposive sampling of three representative provinces (Zhejiang, Henan, Guizhou), followed by quota sampling within each province to enroll participants from tertiary, secondary, and primary care institutions within each province according to predefined targets. US was measured using the Uncertainty Stress Scale and SRH were assessed using the single-item measure. Binary logistic regression was performed to evaluate the association.

**Results:**

High US was reported by 26.5% of participants, and only 48.7% rated their health as good (7.1% excellent, 17.9% very good and 23.7% good). After adjustment, the high US group had an adjusted odds ratio (AOR) of 3.38 (95% CI: 2.81–4.07) for poor SRH compared with the low US group. Working in primary healthcare facilities (AOR = 0.73, 95% CI: 0.59–0.90, tertiary hospitals as referent) and working < 40 h per week (AOR = 0.61, 95% CI: 0.48–0.78, ≥60 h per week as referent) were statistically associated with better SRH, whereas an intermediate professional title was marginally associated with poorer SRH (AOR = 1.31, 95% CI: 1.00–1.70, senior title as the referent). Sensitivity analyses confirmed the robustness of the findings.

**Conclusions:**

High US is significantly associated with poor SRH among Chinese HCPs. This uncertainty stressor may represent a critical psychosocial determinant of self-perceived health in this group. Targeted interventions to mitigate US are warranted to safeguard the wellbeing of HCPs and support the sustainability of healthcare systems.

## Introduction

The physical and mental health of healthcare professionals (HCPs) directly impacts the quality and safety of patient care. Owing to the intrinsic demands of their roles, HCPs operate in persistently high-pressure environments marked by heavy workloads, deep emotional engagement, and considerable clinical decision-making risks (1, 2). Consequently, they constitute a population highly vulnerable to occupational stress and psychological impairment (3). Research reports a wide prevalence of burnout among HCPs, ranging from 18.4 to 94%, with strong correlations observed between burnout and mental health conditions such as anxiety and depression (4–6). In China, ongoing healthcare system reforms, rising public health expectations, and recurrent public health emergencies have collectively led to an increasingly complex and challenging work environment, significantly threatening the overall wellbeing of HCPs (7–9). Thus, systematically monitoring and evaluating the health status of HCPs is not only important for individual wellbeing but also carries significant public health and practical implications.

Accurately assessing HCPs' health requires appropriate measurement tools. Self-rated health (SRH) is defined as an individual's subjective, integrated evaluation of their own health, reflecting a composite of physiological, psychological, and social functioning (10, 11). Numerous studies have confirmed the reliability, validity, and predictive utility of SRH (12). Poor self-rated physical health predicts increased mortality (13), is associated with both fatal and non-fatal cardiovascular events (HR = 1.9; 95% CI: 1.26–2.87) (14), and is linked to elevated risk of chronic kidney disease (HR = 1.278; 95% CI: 1.114–1.465) (15). Compared with objective health indicators, SRH more comprehensively captures an individual's overall perception and functional status (16). For HCPs, who operate in highly complex and stressful environments, SRH can sensitively detect changes in health status, serving as a simple, reliable, and large-scale assessment tool. Given that HCPs often face shift work, occupational exposure, emotional fluctuations, and other special conditions, SRH integrates the combined health effects of these factors, offering unique applicability and advantages in this study.

Among the many factors influencing HCPs' health, stressors have drawn considerable attention. For a long time, researchers have focused on two classic stress-related constructs. The first is occupational stress, which typically arises from external factors such as heavy workload and tense clinician-patient relationships. The second is burnout, a syndrome of emotional exhaustion, depersonalization, and reduced personal accomplishment resulting from prolonged stress (17, 18). However, recent studies suggest that there exists another distinct and pervasive psychosocial stressor, uncertainty stress (US), which has not received sufficient attention among (19). Unlike occupational stress and burnout, US primarily emphasizes the cognitive uncertainty and accompanying psychological tension that individuals experience when facing unpredictable processes, outcomes, or their own coping abilities (20). Chronic exposure to high US is associated with increased anxiety, depression (21, 22), and even self-harm and suicidal ideation (23). In the healthcare setting, US is ubiquitous: diagnostic ambiguity, unpredictable treatment outcomes, complex clinician-patient communication, and ever-changing prevention and control requirements during public health emergencies all make HCPs a highly exposed group (24–26).

However, current research on US has mostly focused on community residents, university students, or addictive behaviors (27–29). For example, Chen et al. observed a correlation between urban residents' US and a higher prevalence of non-communicable diseases (29), and Yang et al. found that nearly one-third (31.1%) of college students reported high US (30). In contrast, large-scale national empirical evidence specifically examining the relationship between US and overall health perception in HCPs remains scarce. Although our previous studies have linked US to depression (21) and sleep quality (22) among HCPs, these outcomes reflect only specific dimensions of mental or physical health. In contrast, SRH provides a holistic assessment, capturing the cumulative burden of US across physical, psychological, and social domains. Examining SRH allows us to evaluate whether the impact of US extends beyond discrete symptoms to affect HCPs‘ perception of their overall health. This issue has not yet been explored in the literature.

Therefore, this study aims to investigate the correlation between US and SRH in Chinese HCPs via a nationwide cross-sectional survey. We hypothesize that elevated US is significantly associated with poorer SRH outcomes. The findings are intended to provide preliminary empirical evidence on the psychosocial pathways linking US to SRH in HCPs and to serve as a reference for future longitudinal studies and the development of health promotion interventions.

## Methods

### Study design and sampling

This nationwide cross-sectional study was conducted among HCPs from September 2022 to January 2023, using a structured questionnaire and self-reporting method. Prior to data collection, all participants were informed online about the procedures regarding informed consent, data anonymization, and confidentiality. The study was approved by the Ethics Committee of Sir Run Run Shaw Hospital, Zhejiang University School of Medicine (Approval No. 2022-0370).

A two-stage sampling strategy was implemented:

Step 1, purposive sampling was used to select three provinces representing distinct socio-economic and geographic profiles: Zhejiang Province (in eastern China with a gross domestic product (GDP) of RMB 7,351.6billion in 2021, ranked 4th nationally) (31), Guizhou Province (in western China with a GDP of RMB 1,958.6 billion in 2021, ranked 22nd nationally) (32), and Henan Province (in central China with a GDP of RMB 5888.7 billion in 2021, ranked 12th nationally) (33).

Step 2, quota sampling within each province identified 3–5 tertiary hospitals (70–90 participants each), 6–8 secondary hospitals (30–50 participants each), and 18–20 primary care institutions (10–30 participants each). Recruitment within institutions utilized either cluster sampling or stratified quota sampling based on department or professional title. At each hospital, two trained coordinators emphasized ethical principles, including confidentiality, and distributed the online questionnaire via WeChat work groups or DingTalk, a common communication platform in China.

The study was aligned with a larger HCPs survey targeting 3,000 respondents. Assuming a prevalence of US of 0.45 and a margin of error of 0.013, the minimum required sample size was calculated as 2,870. We received 2,996 responses, of which 2,976 were valid, resulting in a 99.3% valid response rate.

### Study participants

The survey targeted HCPs who had direct contact with patients in medical institutions at various levels across the eastern, central and western regions of China.

#### Inclusion criteria

HCPs aged between 18 and 65 years, employed at any level or type of medical institution, holding relevant professional qualifications and practice certificates, volunteered to participate in the study and provided electronic informed consent were included in the survey.

#### Exclusion criteria

HCPs who were on authorized or sick leaves for ≥2 weeks during the survey period and who were working as hospital administrators were excluded.

### Study variables

#### Outcome variables

Self-rated health (SRH), which combines subjective perceptions with objective health conditions, provides a robust measure of population health status (12). This study employed SRH to evaluate the health of HCPs in China. Participants responded to the question: “Overall, how would you rate your health?” on a 5-point Likert scale (excellent, very good, good, fair, and poor). This single-item self-rated health measure has been widely used and validated in large-scale epidemiological surveys, demonstrating good predictive validity for mortality and morbidity. Responses were dichotomized into “Good” (excellent, very good, good) and “Poor” (fair, poor) to facilitate clinical interpretation and comparability with prior literature, with “Good” coded as 0 and “Poor” coded as 1, with “Good” group as the reference group. For descriptive analyses, variables retained their original categories; for regression models, the outcome was dichotomized as above.

#### Exposure variables

Uncertainty stress (US) was assessed using the four-item Uncertainty Stress Scale which developed by Professor Yang's team. The scale comprises of four items: current life uncertainty, social change uncertainty, goal achievement uncertainty, and social value uncertainty, each rated on a 5-point Likert scale (1 = never to 5 = frequently), reflecting the respondent's experience over the preceding 4 weeks. Total scores range from 4 to 20, with higher scores indicating greater stress. Based on previous studies (20), participants were categorized into a high US group (score > 12) or a low US group (score ≤ 12). In the present sample, the scale demonstrated good internal consistency (Cronbach's α = 0.81). Additionally, Wu et al. validated the four-item version of this scale in a community-based sample of Chinese adults (34), demonstrating good structural, convergent, and criterion-related validity, further supporting its use in the present study.

#### Covariates

Demographic characteristics (age, gender, marital status, annual income), work-related variables (work district, medical institution level, profession, professional title, years of work experience, weekly working hours) as covariates (Table 1).

**Table 1:** Sociodemographic characteristics of participants stratified by SRH (*n* = 2,976).

		Self-rated health *n* (%)							
Demographic variables	Total	Excellent	Very good	Good	Fair	Poor	χ^2^	Cramer's V	*P*-value
	2,976	211 (7.1)	533 (17.9)	705 (23.7)	1,336 (44.9)	191 (6.4)			
Gender							9.57	0.057	< 0.05
Male	927 (31.1)	83 (39.3)	171 (32.1)	205 (29.1)	403 (30.2)	65 (34)			
Female	2,049 (68.9)	128 (60.7)	362 (67.9)	500 (70.9)	933 (69.8)	126 (66)			
Age (years)							20.98	0.059	< 0.05
18 ~ 29	945 (31.8)	84 (39.8)	180 (33.8)	223 (31.6)	410 (30.7)	48 (25.1)			
30 ~ 39	1,040 (34.9)	49 (23.2)	176 (33)	260 (36.9)	480 (35.9)	75 (39.3)			
40 ~ 65	991 (33.3)	78 (37)	177 (33.2)	222 (31.5)	446 (33.4)	68 (35.6)			
Marital status							15.47	0.051	0.51
Married	2,124 (71.4)	146 (69.2)	370 (69.4)	486 (68.9)	973 (72.8)	149 (78)			
Unmarried	781 (26.2)	64 (30.3)	151 (28.3)	201 (28.5)	329 (24.6)	36 (18.8)			
Other	71 (2.4)	1 (0.5)	12 (2.3)	18 (2.6)	34 (2.5)	6 (3.1)			
Annual income (10,000 RMB)							61.58	0.083	< 0.001
< 5	757 (25.4)	86 (40.8)	157 (29.5)	150 (21.3)	323 (24.2)	41 (21.5)			
5 ~ 9.99	979 (32.9)	68 (32.2)	177 (33.2)	222 (31.5)	434 (32.5)	78 (40.8)			
10 ~ 14.99	634 (21.3)	26 (12.3)	92 (17.3)	174 (24.7)	295 (22.1)	47 (24.6)			
≥15	606 (20.4)	31 (14.7)	107 (20.1)	159 (22.6)	284 (21.3)	25 (13.1)			
District							33.08	0.075	< 0.001
Western	714 (24)	72 (34.1)	134 (25.1)	165 (23.4)	310 (23.2)	33 (17.3)			
Central	903 (30.3)	68 (32.2)	168 (31.5)	182 (25.8)	414 (31)	71 (37.2)			
Eastern	1,359 (45.7)	71 (33.6)	231 (43.3)	358 (50.8)	612 (45.8)	87 (45.5)			
Level of medical institution							29.34	0.07	< 0.001
Primary	1,157 (38.9)	106 (50.2)	237 (44.5)	263 (37.3)	470 (35.2)	81 (42.4)			
Secondary	715 (24)	43 (20.4)	110 (20.6)	175 (24.8)	339 (25.4)	48 (25.1)			
Tertiary	1,104 (37.1)	62 (29.4)	186 (34.9)	267 (37.9)	527 (39.4)	62 (32.5)			
Profession							18.44	0.056	< 0.05
Clinician	1,486 (49.9)	86 (40.8)	264 (49.5)	373 (52.9)	672 (50.3)	91 (47.6)			
Nurse	806 (27.1)	59 (28)	137 (25.7)	171 (24.3)	383 (28.7)	56 (29.3)			
Other	684 (23)	66 (31.3)	132 (24.8)	161 (22.8)	281 (21)	44 (233)			
Professional title							45.64	0.088	< 0.001
Junior	1,328 (44.6)	129 (61.1)	270 (50.7)	299 (42.4)	552 (41.3)	78 (40.8)			
Intermediate	1,084 (36.4)	55 (26.1)	160 (30)	264 (37.4)	522 (39.1)	83 (43.5)			
Senior	564 (19)	27 (12.8)	103 (19.3)	142 (20.1)	262 (19.6)	30 (15.7)			
Years of work experience (years)							19.82	0.058	< 0.05
< 10	1,474 (49.5)	111 (52.6)	27 (51.8)	364 (51.6)	645 (48.3)	78 (40.8)			
10 ~ 19	859 (28.9)	45 (21.3)	135 (25.3)	203 (28.8)	408 (30.5)	68 (35.6)			
≥20	643 (21.6)	55 (26.1)	122 (22.9)	138 (19.6)	283 (21.2)	45 (23.6)			
Working hours (hours/week)							92.37	0.102	< 0.001
< 40	577 (19.4)	71 (33.6)	118 (22.1)	156 (22.1)	205 (15.3)	27 (14.1)			
40 ~ 49	1,098 (36.9)	65 (30.8)	222 (41.7)	266 (37.7)	500 (37.4)	45 (23.6)			
50 ~ 59	530 (17.8)	23 (10.9)	83 (15.6)	108 (15.3)	271 (20.3)	45 (23.6)			
≥60	771 (25.9)	52 (24.6)	110 (20.6)	175 (24.8)	360 (26.9)	74 (38.7)			
US							295.12	0.315	< 0.001
Low	2,186 (73.5)	184 (87.2)	476 (89.3)	576 (81.7)	883 (66.1)	67 (35.1)			
High	790 (26.5)	27 (12.8)	57 (10.7)	129 (18.3)	453 (33.9)	124 (64.9)			

US, uncertainty stress; SRH, self-rated health; RMB, renminbi.

### Statistical analysis

All statistical analyses were performed using SPSS version 26.0 (IBM Corp., Armonk, NY, USA). The analytical procedure comprised the following steps:

#### Descriptive analysis

Sociodemographic and occupational characteristics, US, and SRH were described. Categorical variables were presented as frequencies and percentages, and between-group differences were examined using the chi-square test.

#### Modeling analysis

Multicollinearity among explanatory variables was assessed using the variance inflation factor (VIF). The VIF values ranged from 1.02 to 3.91, indicating no significant multicollinearity among the independent variables. Binary logistic regression was subsequently employed. Model 1 was a univariate analysis without covariate adjustment. Model 2 included dichotomized US as the independent variable, adjusting for sociodemographic and work-related characteristics, to examine its association with SRH. To test robustness, we conducted two sensitivity analyses: (1) treating US as a continuous variable (Model 3) to avoid information loss from dichotomization; and (2) using the median US score as a data-driven cut-off for dichotomization (Model 4), independent of any *a priori* threshold, to test sensitivity to grouping method. Due to the high prevalence of poor SRH in this study, we employed modified Poisson regression with robust error variance to estimate the prevalence ratio (Model 5).

To address potential clustering effects of individuals nested within hospitals, we used a generalized linear mixed model (GLMM) as a sensitivity analysis, with healthcare facilities as random intercepts and regions as fixed effects, to quantify between-facility variation and test the robustness of the main model results.

All tests were two-tailed, with statistical significance set at *p* < 0.05.

## Results

### Sociodemographic characteristics, US and SRH

The sociodemographic profile of the Chinese HCPs is presented in Table 1. Among the 2,976 participants (comprising physicians, nurses, and other HCPs), 26.5% exhibited high US. In terms of SRH, 44.9% described their health as “fair,” 6.4% as “poor,” and only 7.1% as “excellent” (Table 1). SRH significantly differed across most examined subgroups, including gender, age, annual income, region, institution level, profession, professional title, work experience, weekly working hours, and US level (all *p* < 0.05), while no significant difference was observed by marital status (*p* = 0.51) (Table 1). Among variables that showed statistically significant differences, US was moderately associated with SRH (Cramer's *V* = 0.315, *p* < 0.001), while the remaining independent variables exhibited very weak associations with SRH, with Cramer's V values ranging from 0.056 to 0.102.

### Multivariate analysis of US and SRH

Binary logistic regression was performed to examine the association between US and SRH. In the unadjusted model (Model 1), high US was significantly associated with poor SRH (OR = 3.25, 95% CI: 2.95–4.21, *p* < 0.001). After adjusting for sociodemographic and work-related variables (Model 2), the association remained robust: participants experiencing high US had higher odds of reporting poor SRH (AOR = 3.38, 95% CI: 2.81–4.07, *p* < 0.001). Among the covariates, working in a primary care institution (vs. a tertiary hospital; AOR = 0.73, 95% CI: 0.59–0.90) and working fewer than 40 h per week (vs. ≥60 h; AOR = 0.61, 95% CI: 0.48–0.78) were associated with better SRH, while intermediate professional title (vs. senior title; AOR = 1.31, 95% CI: 1.00–1.70) was marginally associated with poorer SRH (Model 2; see Table 2).

**Table 2:** Binary logistic regression analysis of the correlation between US and SRH among HCPs.

Variables	Self-rated health				
	Model 1	Model 2	Model 3	Model 4	Model 5
	OR (95%CI)	AOR (95%CI)	B(95%CI)	AOR (95%CI)	PR (95%CI)
Gender (female as referent)					
Male	0.95 (0.81, 1.11)	1.00 (0.83, 1.19)	0.94 (0.78, 1.13)	0.94 (0.79, 1.13)	1.00 (0.92, 1.08)
Age (years) (40 ~65 as referent)					
18 ~ 29	0.87 (0.73, 1.04)	1.26 (0.87, 1.83)	1.37 (0.94, 2.01)	1.27 (0.88, 1.85)	1.11 (0.94, 1.31)
30 ~ 39	1.06 (0.89, 1.26)	0.91 (0.69, 1.18)	0.93 (0.71, 1.22)	0.96 (0.74, 1.26)	0.96 (0.86, 1.08)
Marital status (other as referent)					
Married	0.87 (0.54, 1.40)	0.85 (0.52, 1.40)	0.89 (0.53, 1.48)	0.83 (0.50, 1.36)	0.92 (0.75, 1.14)
Unmarried	0.68 (0.42, 1.11)	0.67 (0.39, 1.16)	0.70 (0.40, 1.23)	0.68 (0.39, 1.18)	0.83 (0.66, 1.04)
Annual income (10,000 RMB) (≥15 as referent)					
< 5	0.89 (0.72, 1.10)	1.11 (0.79, 1.57)	0.98 (0.69, 1.39)	1.04 (0.74, 1.47)	1.04 (0.90, 1.21)
5 ~ 9.99	1.05 (0.86, 1.29)	1.21 (0.92, 1.59)	1.14 (0.86, 1.51)	1.17 (0.89, 1.54)	1.08 (0.97, 1.22)
10 ~ 14.99	1.12 (0.90, 1.41)	1.13 (0.88, 1.45)	1.08 (0.83, 1.39)	1.10 (0.86, 1.42)	1.06 (0.95, 1.18)
District (eastern as referent)					
Western	0.87 (0.73, 1.05)	0.88 (0.70, 1.11)	0.84 (0.67, 1.07)	0.87 (0.69, 1.10)	0.95 (0.85, 1.05)
Central	1.10 (0.93, 1.30)	0.82 (0.65, 1.03)	0.81 (0.64, 1.02)	0.87 (0.69, 1.10)	0.91 (0.83, 1.01)
Level of medical institution (tertiary as referent)					
Primary	0.80 (0.67, 0.94)^**^	0.73 (0.59, 0.90)^**^	0.70 (0.56, 0.87)^**^	0.75 (0.60, 0.93)^**^	0.86 (0.78, 0.95)^**^
Secondary	1.038 (0.85, 1.24)	0.98 (0.79, 1.22)	0.97 (0.78, 1.21)	1.03 (0.83, 1.28)	0.99 (0.90, 1.09)
Profession (other profession as referent)					
Clinician	1.17 (0.97, 1.40)	1.00 (0.90, 1.35)	1.13 (0.92, 1.39)	1.11 (0.91, 1.36)	1.04 (0.95, 1.15)
Nurse	1.32 (1.08, 1.62)^**^	1.25 (0.99, 1.57)	1.21 (0.95, 1.53)	1.19 (0.94, 1.51)	1.11 (1.00, 1.23)
Professional title (senior as referent)					
Junior	0.84 (0.69, 1.02)	1.02 (0.73, 1.41)	1.05 (0.75, 1.47)	1.00 (0.72, 1.39)	1.01 (0.87, 1.17)
Intermediate	1.18 (0.96, 1.44)	1.31 (1.00, 1.70)^*^	1.32 (1.00, 1.72)^*^	1.26 (0.97, 1.64)	1.12 (1.00, 1.25)^*^
Years of work experience (years) (≥20 years' work experience as referent)					
< 10	0.93 (0.77, 1.11)	0.88 (0.63, 1.23)	0.78 (0.55, 1.10)	0.81 (0.58, 1.14)	0.94 (0.82, 1.09)
10 ~ 19	1.19 (0.97, 1.47)	1.05 (0.80, 1.38)	0.94 (0.71, 1.25)	0.93 (0.71, 1.23)	1.01 (0.90, 1.14)
Working hours (hours/week) (≥60 h/w as referent)					
< 40	0.52 (0.42, 0.65)^***^	0.61 (0.48, 0.78)^***^	0.72 (0.56, 0.93)^*^	0.61 (0.48, 0.78)^***^	0.78 (0.69, 0.88)^***^
40 ~ 49	0.77 (0.64, 0.92)^**^	0.80 (0.65, 0.99)^*^	0.89 (0.72, 1.11)	0.80 (0.65, 0.98)^*^	0.90 (0.83, 0.99)
50 ~ 59	1.15 (0.92, 1.44)	1.15 (0.91, 1.46)	1.21 (0.94, 1.54)	1.14 (0.89, 1.44)	1.05 (0.96, 1.15)
US (Low US as referent)					
High	3.25 (2.95, 4.21)^***^	3.38 (2.81, 4.07)^***^	–	3.37 (2.89, 3.94)^***^	1.63 (1.52, 1.74)^***^
US score	–	–	1.42 (1.36, 1.48)^***^	–	–

^*^p < 0.05, ^**^p < 0.01, ^***^p < 0.01.

Model 1: unadjusted binary logistic regression was conducted.

Model 2: adjusted binary logistic regression was performed, incorporating dichotomous US as the predictor and SRH as the outcome, while controlling for sociodemographic, work-related, and psychosocial covariates.

Model 3: To assess robustness, US was entered as a continuous variable in the adjusted model specified in Model 2.

Model 4: A sensitivity analysis was carried out by redefining US as a dichotomous variable (split at the median total score) within the same adjusted logistic regression framework as Model 2.

Model 5: Modified Poisson regression was used to estimate prevalence ratios, with dichotomized US as the predictor, SRH as the outcome, and adjusting for socioeconomic, work-related, and psychosocial covariates.

AOR, adjusted OR; HCPs, healthcare professional; US, uncertainty stress; RMB, renminbi; PR, prevalence ratios.

### Sensitivity analysis

To assess the robustness of the findings, we performed sensitivity analyses. First, US was entered as a continuous variable in Model 3. Each one-point increase in US was associated with a 1.42-fold increase in the odds of reporting poor SRH (*B* = 1.42, 95% CI: 1.36–1.48, *p* < 0.001), supporting a dose-response relationship. Second, US was dichotomized by the median into high and low groups (Model 4). The high US group remained significantly associated with poorer SRH (AOR = 3.37, 95% CI: 2.89–3.94, *p* < 0.001), which was nearly identical to the primary analysis result (AOR = 3.38), indicating that the grouping method had a negligible impact on the conclusion. The direction and significance of the covariates (working in a primary care facility and working < 40 h/week) in Models 3 and 4 were consistent with those in Model 2 (see Table 2). Third, given that the high prevalence of poorer SRH may lead to overestimation of the association strength when using OR, we further applied modified Poisson regression to estimate prevalence ratios (PRs; Model 5). The results showed that the high US group remained significantly associated with poorer SRH (PR = 1.63, 95% CI: 1.52–1.74, *p* < 0.001). Although this PR was smaller than the OR from the primary analysis, the association remained significant and did not compromise the robustness of the conclusions. The direction of PR estimates for other covariates was consistent with the Model 2 and 3 analysis, e.g., primary care workers: PR = 0.86 (95% CI: 0.78–0.95); intermediate professional title: PR = 1.12 (95% CI: 1.00–1.25); working < 40 h/week: PR = 0.78 (95% CI: 0.69–0.88).

The GLMM results indicated that the between-hospital variance of the random intercept was 0.033 (SE = 0.114), with a corresponding intraclass correlation coefficient of only 0.008, which was not statistically significant. The variance estimate was not statistically significant, suggesting that the clustering effect at the hospital level was negligible. Therefore, ordinary logistic regression was retained as the primary model to ensure interpretability of the results.

## Discussion

To our best knowledge, this is the first national multicenter cross-sectional study addressing the research gap regarding the association between US and SRH among HCPs in China. The research sample covered HCPs from different economic development levels and geographic regions, as well as various tiers of medical institutions across the country. The results demonstrate that higher US is significantly associated with poorer SRH. Specifically, more than half of the participants reported poor SRH, and over one-quarter experienced high US. These findings highlight the urgency of developing precise interventions aimed at mitigating US in this professional group.

The study observed that only 48.7% of HCPs reported good SRH, a proportion significantly lower than that observed in the general adult population (81.7%) (35) and in middle-aged and older adults (79.4%) (13). This discrepancy may reflect the high sensitivity of HCPs to the physical and mental demands. For instance, Esperanza et al. noted that sleep deprivation during shifts can induce manic symptoms among HCPs (36), while additional work pressures have led one-quarter to one-third of physicians to report a deterioration in their mental health status (37). The poor SRH rate (51.3%) among HCPs in this study was lower than the reported job burnout rates (57.7%−71.3%) (6, 38), suggesting that occupational stress may not only lead to psychological burnout but also prompt negative self-assessments of overall health.

Among the factors influencing SRH, working in a primary medical institution and having shorter weekly duty hours are statistically correlated with better SRH among HCPs, compared with covariates such as gender, age, income, and region. This association may be explained by several factors. Primary care practitioners generally experience a steadier workflow, limited exposure to acute or complex clinical cases, and more stable patient-provider interactions (39). While excessively long working hours may lead to fatigue accumulation, moderate workloads can help alleviate emotional exhaustion, thereby improving SRH. This study found a marginally significant association between intermediate professional titles and poorer SRH, which is inconsistent with Jin et al.‘s finding that higher title predicted worse health (40). This discrepancy may be attributed to differences in population characteristics and health assessment methods, or may be influenced by unmeasured confounding factors. We speculate that individuals with intermediate professional titles are at a critical stage of career development, bearing heavy clinical workloads and performance pressure, and tend to form negative self-evaluations when comparing their health status with that of senior colleagues. However, due to the cross-sectional design of this study, this exploratory finding requires validation by future longitudinal research, which should also incorporate more comprehensive measures of occupational stress to elucidate the underlying mechanisms.

This study reported a 26.5% prevalence of high US among HCPs, which is lower than the 31.1% observed in Chinese university students (30) and diverges from findings among urban Chinese residents (41). The difference may be attributable to distinct study populations and the specific nature of the stressors involved, as well as the distributional differences of US across populations in different regions (42). For HCPs, US is notably occupation-specific and persistent (43). In addition to individual, institutional, and societal factors, professional dimensions such as diagnostic ambiguity, treatment decision-making risks, the complexity of doctor-patient communication, and pressures from responding to public health emergencies constitute long-term, high-intensity sources of US exposure for this group (44). Although the prevalence of high-level US among HCPs is lower than that among the general public (29) or student groups (30), the frequency and intensity of stressors in HCPs are highly context-embedded. Moreover, these data were collected during the late COVID-19 pandemic, when changing infection-control policies, work pressure, risk perception, and public health emergency responses may have amplified US, potentially leading to more severe cumulative health effects.

Our study further demonstrates that, even after adjusting for factors such as working hours and institution type, US remained significantly associated with SRH among HCPs (AOR = 3.38). Compared with traditional stressors such as occupational stress, burnout, and workload, US exhibited an additional independent effect. This finding aligns with Zhu et al.‘s early-pandemic observation in the general population (45). This independent effect may stem from the unique nature of US, namely uncertainty about the future, unpredictability of outcomes, and doubt about one's own coping abilities. This cognitive-level uncertainty, which differs from other stressors dominated by external load or emotional exhaustion, is more likely to trigger persistent vigilance and a sense of loss of control, thereby eroding individuals' confidence in their own health. These findings suggest that US is not merely a transient psychological response, but rather a salient determinant of overall health evaluation in this population, and its strength of association with SRH may exceed that of other common stressors. Regarding potential pathways, the literature suggests that US may affect health through neuroendocrine mechanisms including sustained activation of the hypothalamic-pituitary-adrenal axis and cortisol dysregulation (46, 47). Other studies indicated that US may impair prefrontal cortex function (48), which in turn is associated with burnout, anxiety, depression, and sleep disturbances (49). Such psychological states not only directly lower subjective health ratings but may also be accompanied by unhealthy behaviors (e.g., smoking, drinking, physical inactivity) that further compromise objective health (50). However, these proposed mechanisms require experimental or longitudinal validation.

Based on the above cross-sectional observational study, we propose the following preliminary practical recommendations. At the public health policy level, unified risk communication mechanisms should be established, and psychological stress should be included in occupational health monitoring. Existing studies have shown that optimizing risk communication strategies is beneficial for alleviating US (51, 52). At the institutional level, US management training (e.g., mindfulness-based stress reduction, cognitive-behavioral therapy) and a tiered psychological support system comprising emergency psychological aid teams and anonymous counseling hotlines may be considered. Multiple studies have shown that social support can alleviate US (53) and reduce emotional exhaustion, and maintaining social connections is crucial for alleviating psychological distress (54, 55). At the individual level, HCPs should enhance their awareness of the US and proactively learn coping skills while utilizing available support resources. Future interventional studies could further investigate effective intervention strategies targeting uncertainty.

This study has several limitations. Firstly, the cross-sectional design cannot establish a causal relationship. Future longitudinal studies are needed to track individuals and clarify the temporal relationship between US and SRH. Secondly, all variables were self-reported, which may introduce recall bias and social desirability bias; future research could incorporate objective indicators to improve assessment accuracy. Thirdly, a combination of purposive sampling and cluster sampling was used, which may introduce selection bias within institutions and potentially limit the generalizability of the findings to all HCPs nationwide. Although we achieved broad geographic and institutional coverage, heterogeneity within and across provinces may still exist. Fourthly, potential confounders such as job burnout and social support were not measured, which may affect the estimation of the associations.

## Conclusion

This study demonstrates that higher US is significantly associated with poorer SRH among HCPs in China, and this association remains robust across different models. These results underscore US as a critical psychosocial factor influencing health perceptions in this professional group. Multilevel interventions, such as organizational support, psychological interventions, and policy optimization, may be implemented to alleviate US among HCPs. Such interventions may improve their physical and mental wellbeing and support the long-term sustainability of the healthcare system.

## Data Availability

The datasets presented in this article are not readily available as our ethical requirements prohibit the public disclosure of participants' data. However, they can be obtained from the corresponding author upon reasonable request.
